# The Singaporean public beliefs about the causes of mental illness: results from a multi-ethnic population-based study

**DOI:** 10.1017/S2045796017000105

**Published:** 2017-04-03

**Authors:** S. Pang, M. Subramaniam, S. P. Lee, Y. W. Lau, E. Abdin, B. Y. Chua, L. Picco, J. A. Vaingankar, S. A. Chong

**Affiliations:** Research Division, Institute of Mental Health, Singapore

**Keywords:** Mental health, mental illness stigma, multicultural, population survey, social distance

## Abstract

**Aims.:**

To identify the common causal beliefs of mental illness in a multi-ethnic Southeast Asian community and describe the sociodemographic associations to said beliefs. The factor structure to the causal beliefs scale is explored. The causal beliefs relating to five different mental illnesses (alcohol abuse, depression, obsessive-compulsive disorder (OCD), dementia and schizophrenia) and desire for social distance are also investigated.

**Methods.:**

Data from 3006 participants from a nationwide vignette-based study on mental health literacy were analysed using factor analysis and multiple logistic regression to address the aims. Participants answered questions related to sociodemographic information, causal beliefs of mental illness and their desire for social distance towards those with mental illness.

**Results.:**

Physical causes, psychosocial causes and personality causes were endorsed by the sample. Sociodemographic differences including ethnic, gender and age differences in causal beliefs were found in the sample. Differences in causal beliefs were shown across different mental illness vignettes though psychosocial causes was the most highly attributed cause across vignettes (endorsed by 97.9% of respondents), followed by personality causes (83.5%) and last, physical causes (37%). Physical causes were more likely to be endorsed for OCD, depression and schizophrenia. Psychosocial causes were less often endorsed for OCD. Personality causes were less endorsed for dementia but more associated with depression.

**Conclusions.:**

The factor structure of the causal beliefs scale is not entirely the same as that found in previous research. Further research on the causal beliefs endorsed by Southeast Asian communities should be conducted to investigate other potential causes such as biogenetic factors and spiritual/supernatural causes. Mental health awareness campaigns should address causes of mental illness as a topic. Lay beliefs in the different causes must be acknowledged and it would be beneficial for the public to be informed of the causes of some of the most common mental illnesses in order to encourage help-seeking and treatment compliance.

## Introduction

Stigma is one of the biggest barriers to help-seeking for those with mental illness (Corrigan & Watson, 2002). It is related to a lower level of mental health literacy (MHL) or understanding of the many facets of mental illnesses (Jorm, 2000). Attributions or beliefs about the causes of mental illness is one aspect of MHL and stigma. Misconceptions about the causes of mental illness can increase stigma and desire for social distance towards the mentally ill and prevent help-seeking behaviour, prolonging the duration of untreated illness (Corrigan & Watson, 2002; Corrigan, 2004; Reavley & Jorm, 2014*a*). People may also avoid seeking help for mental illness or seek help from inappropriate sources based on what they believe is causing their symptoms. Chen & Mak (2008) suggested that European Americans and Chinese Americans more likely seek help from mental health professionals compared with Hong Kong Chinese and Mainland Chinese due to different lay beliefs about the causes of mental illness. Hence, causes of mental illness is one of the important topics which should be addressed in a culture-appropriate manner when educating lay people about mental illness in order to reduce stigma and help those with mental illness receive the help they need.

Although misconceptions of the causes of mental illness are prevalent worldwide, there appear to be cultural differences in causal beliefs of mental illness. Cultural beliefs play a significant role in determining the explanatory models of illness (Kleinman, 1980) and early research into cross-cultural attributions of mental illnesses suggested that there were multiple, separate explanations that people tend to endorse, including western concepts of physiology (e.g., Chemical imbalances in brain, genetics), non-western concepts of physiology (e.g., Traditional Chinese beliefs of a body out of balance or harmony), stress and supernatural causes (Maurice, 1990). Research generally suggests that Westerners have more biological and psychological beliefs while non-Westerners have sociological and theological explanations of mental illness (Nakane *et al*. 2005; Furnham & Telford, 2011). An article comparing Australian and Japanese lay beliefs about the causes of mental illness revealed that ‘social and personal vulnerability causes were commonly endorsed’ in both countries (Nakane *et al*. 2005). However, the researchers found that Japanese were more likely to endorse ‘weakness of character’ as a cause while Australians more likely to believe in physiological causes (infections, allergies and genetics).

A number of previous studies have explored non-western perceptions of mental illness. In a study conducted in Malaysia, 53% of Malay psychiatric patients believed that their illness had supernatural causes (Razali *et al*. 1996). UK Arabs had stronger beliefs in supernatural and non-western physiological causal beliefs than Caucasians (Hamid & Furnham, 2013). Similarly, British Indian immigrants showed stronger beliefs in supernatural causes than their British Caucasian counterparts (Jobanputra & Furnham, 2005).

Interestingly, Bhikha *et al*. (2015) found that 55.5% of British South Asians in their study endorsed supernatural causes of psychosis but the majority of them (77.7%) had a dual explanatory model – endorsing both supernatural and biological causes – which was reflected in their help-seeking behaviour through a combination of prescribed medication and traditional healing. It appeared that the respondents were able to hold supposedly conflicting beliefs simultaneously, suggesting that lay beliefs are multidimensional and inform help-seeking choices. While other studies including respondents of Asian ethnicities do suggest a tendency to endorse supernatural or non-western physiological causes, it is hard to say that such beliefs are mutually exclusive. Cultural differences and changing cultural perspectives due to globalisation thus have a strong influence on individuals’ views towards mental illness and its causes.

Causal beliefs not only affect help-seeking choices, but are related to behaviours such as stigma and social distancing towards the mentally ill. For example, a national survey of the Australian public found that belief in weak or nervous personality as the cause of mental illness was associated with personal stigma, perceived stigma and desire for social distance towards those with depression, schizophrenia, social phobia and post-traumatic stress disorder (Reavley & Jorm, 2014*a*). Population surveys conducted in Germany found endorsement of biogenetic causes for depression and schizophrenia to be associated with greater desire for social distance (Dietrich *et al*. 2006; Schomerus *et al*. 2014). However, lower social distancing from those with schizophrenia was associated with the belief in current stress as a cause of the illness (Schomerus *et al*. 2014). These studies show that causal beliefs can not only affect how people seek treatment if they have mental illness, but their attitudes and levels of tolerance towards those who are mentally ill.

The different methodologies used by previous studies make cross-cultural comparisons difficult. Razali *et al*. (1996) recruited a small sample of 153 psychiatric patients and used a 20-item checklist to measure beliefs. Hamid & Furnham (2013) recruited students via emails and special interest groups through advertisements on social networking sites. They used the Mental Distress Explanatory Model Questionnaire (MDEMQ, Eisenbruch, 1990) to measure causal beliefs and attitudes towards both seeking psychological help and those with mental illness. The different measures used, sample size and non-representative sampling methods make it hard to generalise the findings to other communities or track changes in belief over time. Furthermore, most previous studies have focused on causal beliefs of schizophrenia and depression (Nakane *et al*. 2005; Loo & Furnham, 2012, 2013) or only addressed mental illness in general without specifying particular disorders (Sheikh & Furnham, 2000; Hamid & Furnham, 2013).

However, some recent studies around the world have used a methodology similar to that of Jorm *et al*.’s (1997) studies for the Australian national survey of MHL. These studies include those conducted in Australia, Hong Kong and Japan (Nakane *et al*. 2005; Lam, 2014; Reavley & Jorm, 2014*a*) allowing easy cross-cultural comparison of findings. This method involves a vignette-based approach of examining MHL where participants read a vignette describing a character with mental illness and then they are administered scales to measure their recognition, attitudes and beliefs about mental illness. This approach can and has been used to explore MHL and its components, which include causal beliefs, stigma and social distance in less studied populations such as those in Southeast Asia.

Singapore is a multi-ethnic island city-state in Southeast Asia with a population of 5.61 million in 2016. The population consists of three main ethnic groups, Chinese (74.3%), Malay (13.4%) and Indian (9.1%), while 3.2% are of other ethnic groups (Population Trends, 2016). A national mental health survey of the population conducted in 2009 found large treatment gaps in those with mental disorders (Chong *et al*. 2012*b*). Only 31.7% of people with mental disorders had sought help with 15.7% visiting mental health providers, 8.4% approached general practitioners and 7.6% seeking help from religious/spiritual advisors or other healers. The disorder with the largest treatment gap was alcohol abuse (96.2%) followed by obsessive compulsive disorder (OCD, 89.8%). Stigma towards mental illness and causal beliefs may explain their help-seeking behaviours. Indeed, one study of Chinese Singaporean youths found five main explanations of mental disorders endorsed by Christian, Chinese religionist and free-thinking youths (Mathews, 2011). The researcher found two psychological (humanistic and cognitive-behavioural), one physiological (including Asian physiological explanations) and two supernatural (karmaic, Asian religious beliefs and classical religious beliefs) factors. Another study of local youths showed that belief in physiological causation was associated with preference for Traditional Chinese Medicine physicians and medical doctors, while belief in psychosocial causes was associated with preference for mental health professionals (Lee, 2008). While these two local studies revealed some of the causal beliefs of mental illness, a significant proportion of the Singaporean population, including adults, Malays and Indians are underrepresented. Understanding the causal beliefs of mental illness may elucidate the sociodemographic and cultural nuances that may have played a role in the significant treatment gap observed in Singapore.

Thus, this paper aims to explore the causal beliefs endorsed by four ethnic groups (Chinese, Malay, Indian and Others) in a nationally representative sample from the Southeast Asian country of Singapore. The study replicates the methodology, which was previously used to compare public causal beliefs in Australia and Japan (Nakane *et al*. 2005; Reavley & Jorm, 2014*a*). Next, we aim to compare these groups on their beliefs of the causes of five mental disorders: depression, schizophrenia, OCD, dementia and alcohol abuse. These disorders were chosen for three main reasons. First, these disorders were previously explored in local epidemiological studies and were identified as common disorders in Singapore – major depressive disorder, alcohol abuse and OCD being the top three most common disorders and dementia having a prevalence of 10% in the older adult population (Chong *et al*. 2012*c*; Subramaniam *et al*. 2015). Second, these disorders were associated with large treatment gaps (Chong *et al*. 2012*a*, *b*). Third, the disorders chosen address the knowledge gap in MHL for disorders other than depression and schizophrenia, while also providing comparable data on cultural differences in the causal beliefs of depression and schizophrenia. Last, the paper investigates the relationship between causal beliefs of mental illness and social distance.

## Method

Data were collected from 3006 Singapore residents aged 18–65 years old as part of Mind Matters: A Study of Mental Health Literacy, the first nation-wide study on MHL in Singapore conducted in 2014. A disproportionate stratified sampling design by age and ethnicity groups was implemented for the study. The sample was derived using the sampling frame from an administrative database in Singapore that maintains the names, sociodemographic details and household addresses of all citizens, permanent residents and foreigners in Singapore. The mean age of the respondents was 40.9 years and 50.9% were males. The majority of respondents were Chinese (74.7%), followed by Malays (12.8%), Indians (9.1%) and other ethnic groups (3.3%).

Respondents participated in a face-to-face survey conducted by a trained lay interviewer in their preferred language (English, Mandarin, Malay or Tamil). One of the five vignettes describing a person with either depression, OCD, alcohol abuse, schizophrenia or dementia was randomly read to each participant. They were then asked questions relating to causal beliefs of mental illness, stigma and social distance. Data on MHL and sociodemographic details (age, gender, ethnicity, educational level and employment status) were also collected. All scales were translated, cognitively tested and modified for understandability for the local population. Written informed consent was obtained from all respondents who were 21 years and above as well as from parents/guardians of participants who were aged 18–20 years. The study yielded a response rate of 71%. More details on the study methodology are outlined by Chong *et al*. (2016).

For the purposes of this analysis, data were drawn from mainly two questionnaires, the causal beliefs about mental illness scale and social distance scale.

## Measurements

### Causal beliefs about mental illness (Reavley & Jorm, 2014*b*)

The scale consisted of ten items where participants were asked to rate the cause of the problem in the vignette on a 5-point scale from ‘Very likely’ to ‘Very unlikely’. Items included ‘a virus or other infection’, ‘an allergy or reaction’, ‘everyday problems such as stress, family arguments, difficulties at work or financial difficulties’, ‘the recent death of a close friend or relative’, ‘some recent traumatic event such as a severe traffic accident’, ‘childhood problems such as being badly abused, losing one or both parents when young or coming from a broken home’, ‘inherited or genetic or run in the family’, ‘spirit possession, supernatural causes or black magic’, ‘being a nervous person’ and ‘having a weak character’.

### Social distance (Link *et al*. 1999)

This scale consists of five questions pertaining to how willing participants would be to have contact with the person in the vignette (1 = definitely willing, 4 = definitely unwilling). Participants were asked how willing they would be to (1) move next door to the person in the vignette, (2) spend an evening with the person, (3) make friends with the person, (4) start working closely with the person and (5) have the person marry into their family. Scores are summed and higher scores indicate greater desire for social distance.

### Analysis

All estimates were weighted to adjust for over-sampling and post-stratified for age and ethnicity distributions between the survey sample and the Singapore Resident population in the year 2012. Factor analysis was conducted to explore the factor structure of the causal beliefs about mental illness scale. Answers to the questions on causal beliefs were dichotomised and exploratory factor analysis (EFA) was conducted in MPLUS version 6 using Promax rotation solution to allow correlations between factors. Prior to EFA, the Kaiser–Meyer–Olkin measure of sampling adequacy and Bartlett's test of sphericity were calculated using SPSS software to determine the suitability of the data for EFA. The factor loading was set as 0.3. Several criteria were used in revised analyses to determine the number of factors such as eigenvalue-based procedures including number of eigenvalues >1.0 and scree plot, pattern of loadings on each factor (i.e., number of non-loading or double-loading items) and interpretability of each solution. The internal reliability (Cronbach's alpha) for each factor was calculated as well. Following factor analysis, multiple logistic regressions using the ‘Enter’ method were conducted to explore sociodemographic predictors of causal beliefs and its relationship with vignette and social distance. All causal belief factors (physical, personality and psychosocial causes) were treated as dependent variables while all sociodemographic variables (age, gender, ethnicity, marital status, employment and monthly income status), vignette type and social distance scores were treated as independent variables. Three series of multiple logistic regression analyses were run separately for each of the causal belief factors to estimate the effects of sociodemographic variables, vignette type and social distance. Reference categories used were female gender, Chinese ethnicity, 18–34 years of age, married status, employed, less than $2000 income, university level education and alcohol abuse. The regression analysis was analysed using SAS version 9.3. All statistically significant results were reported at *p* ≤ 0.05.

## Results

### Factor analysis

The Kaiser–Meyer–Olkin test yielded a value of 0.68, indicating strong partial correlations between the variables after controlling for all other variables. The Bartlett's test of sphericity was statistically significant (*χ*^2^ = 2566.233, degrees of freedom (df) = 45, *p* value <0.001) indicating that both tests supported evidence that EFA could be applied to these data.

The eigenvalues and scree plot on all ten items of the scale suggested that three or four factor models were potential solutions. Initial EFA yielded three factors accounting for 49.7% of the variance (22.9, 14.6 and 12.2%, respectively). The first factor consisted of ‘everyday problems such as stress, family arguments, difficulties at work or financial difficulties’, ‘recent death of a close friend or relative’, ‘recent traumatic event such as a severe traffic accident’ and ‘childhood problems such as being badly treated or abused, losing one or both parents when young or coming from a broken home’. The second factor consisted of ‘being a nervous person’ and ‘having a weak character’, while the third factor consisted of both supernatural and biological attributions of mental illness – ‘a virus or other infection’, ‘allergy or reaction’ and ‘spirit possession, supernatural causes or black magic’.

A closer inspection revealed that the interrelations between ‘spirit possession, supernatural causes or black magic’ with ‘a virus or other infection’ and ‘allergy or reaction’ items were 0.138 and 0.069 (online Supplementary Table 1), respectively. As the ‘inherited or genetic’ item loaded onto all three factors and the inter-item correlation between ‘spirit possession, supernatural causes or black magic’ with other items within factor 3 were relatively weak, both items were removed.

Subsequently, the EFA was rerun with the eight remaining items suggesting that three factors accounted for 59.45% of the variance. Table 1 shows the results of the factor loadings of the eight items. The factor loadings within each factor ranged from 0.38 to 0.78, respectively. The three extracted factors were subsequently selected and labelled – factor 1 (two items) was named ‘physical causes’; factor 2 (two items) was named ‘personality causes’ and factor 3 (four items) was named ‘psychosocial causes’ (Table 1). The Cronbach's alpha for the physical, personality and psychosocial causes were 0.641, 0.649 and 0.557, respectively.

**Table 1.** Results of the factor loadings of the eight items of causal beliefs

Item	Factor 1	Factor 2	Factor 3
	Physical	Personality	Psychosocial
A virus or other infection	**0.72**	−0.10	0.04
An allergy or reaction	**0.59**	0.08	−0.08
Everyday problems such as stress, family arguments difficulties at work or financial difficulties	−0.01	0.13	**0.38**
The recent death of a close friend or relative	−0.03	−0.12	**0.77**
Some recent traumatic event such as a severe traffic accident	0.04	−0.02	**0.56**
Childhood problems such as being badly abused, losing one or both parents when young or coming from a broken home	0.03	0.24	**0.37**
Being a nervous person	0.03	**0.53**	−0.03
Having a weak character	−0.06	**0.78**	−0.06

Values >0.3 are highlighted in bold.

### Sociodemographic differences

Results of the multiple logistic regression analyses are shown in Table 2. Those aged 35–49 years were less likely to endorse psychosocial causes than the younger (18–34 years) age group (OR 0.321). Males (*v*. females, OR 1.524), homemaker/student/retired (*v*. employed, OR 0.683), those with income of $2000 and above (*v*. below $2000, OR ranged from 0.176 to 0.627) were less likely to endorse physical causes while never married individuals *v*. married individuals (OR 1.515) and those with primary level education (OR 2.076) and secondary education (OR 1.812) were more likely to endorse physical causes than those with university education. Males (*v*. females, OR 0.704) and Malays (*v*. Chinese, OR 0.748) were less likely to endorse personality causes while those with a polytechnic diploma (post-secondary diploma) were more likely to endorse personality causes than those with university education (OR 1.743).

**Table 2.** Results of logistic regression for psychosocial, physical and personality causes

	Psychosocial				Physical				Personality			
	OR	95% CI		*p*-value	OR	95% CI		*p*-value	OR	95% CI		*p*-value
Gender												
Female	Reference				Reference				Reference			
Male	0.97	0.46	2.05	0.934	1.52	1.21	1.92	**<0.0001**	0.70	0.52	0.95	**0**.**022**
Ethnicity												
Chinese	Reference				Reference				Reference			
Malay	0.69	0.33	1.45	0.330	0.93	0.75	1.16	0.506	0.75	0.57	0.99	**0.040**
Indian	0.56	0.28	1.12	0.101	0.90	0.72	1.11	0.323	0.86	0.66	1.13	0.283
Others					0.80	0.36	1.77	0.586	0.43	0.16	1.14	0.090
Age group												
18–34 years	Reference				Reference				Reference			
35–49 years	0.32	0.13	0.82	**0.018**	1.32	0.95	1.84	0.098	0.86	0.58	1.29	0.471
50–65 years	0.70	0.20	2.42	0.568	1.27	0.89	1.82	0.189	0.92	0.58	1.45	0.710
Marital status												
Married	Reference				Reference				Reference			
Never married	0.80	0.34	1.88	0.605	1.52	1.12	2.05	**0.008**	0.89	0.61	1.31	0.553
Divorced/separated	0.37	0.09	1.46	0.156	0.68	0.35	1.33	0.263	0.96	0.45	2.09	0.926
Widowed	0.92	0.18	4.70	0.919	0.44	0.17	1.18	0.103	0.85	0.30	2.46	0.767
Employment status												
Employed	Reference				Reference				Reference			
Homemaker/student/retired	0.87	0.30	2.53	0.792	0.68	0.50	0.94	**0.019**	0.90	0.60	1.33	0.592
Unemployed	0.78	0.12	5.03	0.796	0.93	0.53	1.62	0.791	1.61	0.69	3.74	0.269
Monthly income (SGD)												
<SGD $2000	Reference				Reference				Reference			
2000–3999	0.65	0.21	2.00	0.453	0.63	0.47	0.84	**0.002**	1.23	0.84	1.82	0.286
4000–5999	0.79	0.15	4.27	0.786	0.43	0.28	0.65	**<0.0001**	1.19	0.72	1.98	0.501
6000–9999	0.36	0.06	2.20	0.266	0.42	0.24	0.73	**0.002**	1.44	0.73	2.84	0.295
>10 000	1.14	0.16	8.01	0.893	0.18	0.08	0.38	**<0.0001**	2.12	0.85	5.28	0.107
Education level												
No formal	0.33	0.04	2.68	0.297	1.73	1.00	3.01	0.050	1.98	0.93	4.22	0.076
Primary	0.16	0.03	1.03	0.054	2.08	1.18	3.64	**0.011**	1.57	0.73	3.35	0.245
Secondary	0.96	0.17	5.35	0.959	1.81	1.06	3.10	**0.030**	0.99	0.51	1.95	0.985
O/N Level	0.53	0.11	2.61	0.433	1.25	0.85	1.84	0.249	1.27	0.78	2.04	0.337
A Level	1.52	0.27	8.50	0.633	0.72	0.38	1.35	0.301	1.27	0.62	2.58	0.512
Polytechnic	0.71	0.17	3.02	0.643	1.15	0.78	1.68	0.486	1.74	1.03	2.95	**0.039**
Other Diploma	1.17	0.36	3.80	0.795	1.16	0.78	1.72	0.459	1.24	0.77	1.99	0.381
University	Reference				Reference				Reference			
Vignette												
Dementia	0.85	0.22	3.34	0.819	1.23	0.85	1.77	0.266	0.55	0.36	0.83	**0.005**
Depression	1.83	0.31	10.95	0.507	2.03	1.41	2.92	**<0.0001**	1.62	0.99	2.65	0.056
OCD	0.21	0.07	0.65	**0.007**	2.26	1.58	3.24	**<0.0001**	1.18	0.73	1.91	0.505
Schizophrenia	0.93	0.21	4.12	0.928	1.42	1.01	2.02	**0.047**	1.10	0.70	1.72	0.684
Alcohol abuse	Reference				Reference				Reference			
Social distance	0.89	0.42	1.87	0.752	1.03	0.84	1.27	0.748	1.03	0.78	1.36	0.825

Significant values are highlighted in bold.

### Causal attributions across vignettes

The percentage of respondents who endorsed each causal attribution across vignettes is shown in Table 3. Psychosocial causes was the most highly attributed cause across vignettes (endorsed by 97.9% of respondents), followed by personality causes (83.5%) and physical causes (37%).

**Table 3.** Percentage of participants who endorsed causal attribution of mental illness by vignette

Causal attribution of mental illness	%
Psychosocial causes	
Alcohol abuse	98.4
Dementia	98.6
Depression	99.4
OCD	92.5
Schizophrenia	98.6
Total	97.9
Personality causes	
Alcohol abuse	83.4
Dementia	73.2
Depression	89.1
OCD	86.3
Schizophrenia	85.0
Total	83.5
Physical causes	
Alcohol abuse	28.8
Dementia	32.1
Depression	43.1
OCD	44.9
Schizophrenia	35.9
Total	37.0

Participants who had read the OCD vignette were less likely to endorse psychosocial causes (OR 0.211) than those who read the alcohol abuse vignette. Those who had read the vignette describing a person with dementia were significantly less likely to endorse personality causes (OR 0.546). The depression vignette was associated with personality causes (OR 1.598, *p* < 0.05). The physical factor was more likely to be endorsed for the depression (OR 2.029), OCD (OR 2.259) and schizophrenia vignettes (OR 1.42459).

### Social distance

No associations between causal beliefs and social distance were found.

## Discussion

Overall, the factor analysis yielded three factors for causal beliefs of mental illness after the removal of two items. Correlations between causal beliefs were found between sociodemographic factors and the different types of mental illness. However, social distance was not found to be related with causal beliefs.

The factor structure of causal beliefs is similar to that of previous studies. Reavley & Jorm (2014*a*) defined four factors: ‘physical causes’, ‘psychosocial causes’, ‘biogenetic causes’ and ‘weak or nervous personality causes’. In our study, physical, psychosocial causes and personality causes were loaded with the same items. But as the ‘inherited or genetic’ item was loaded onto all three factors, it was removed and our analysis did not produce a ‘biogenetic cause’ factor. The ‘supernatural causes’ item was not used by Reavley & Jorm (2014*a*) and was removed from further analysis due to weak inter-item correlation in the other factors.

The removal of these two items may be because they were the only items of their type. Reavley & Jorm (2014*a*) had one more item – ‘these sorts of problems are caused by a chemical imbalance in the brain’ – that formed the two item biogenetic factor with the ‘inherited or genetic’ item in their study. The development and inclusion of more items concerning biogenetic causes and supernatural causes would be beneficial for use in the local population. This is especially true as supernatural or religious beliefs can affect help-seeking behaviours, for example some may disregard mental health professionals as an appropriate avenue for help as they feel mental professionals disregard religious values and thus prefer to approach traditional healers for treatment (Al-Krenawi & Graham, 2000; Bhikha *et al*. 2015). Indeed, 7.6% of people with mental illness in Singapore reported seeking help from religious/spiritual advisors or other healers (Chong *et al*. 2012*b*) – a significant proportion, which could not be captured with the causal beliefs scale. As spirituality is a key aspect of positive mental health in the local population (Vaingankar *et al*. 2011), engaging traditional healers and religious leaders to increase mental health literacy is important (Bhikha *et al*. 2015). Engaging these key avenues may not only reduce the treatment gap but also reduce stigma and provide social support for those with mental illness. Traditional healers and religious advisors can help identify those with mental illness and encourage them to seek help, while also providing the moral support that spirituality gives to those who are religious. Despite the fact that supernatural beliefs did not emerge as a distinct factor of causal belief of mental illness in this study, it is an important topic to be further researched in the multi-ethnic population of Singapore where many have differing beliefs.

Those of Malay ethnicity were significantly less likely to endorse personality causes than Chinese in our study. Cultural differences between Chinese and Malays were found in a previous study conducted in Malaysia where Malays endorsed religious attributions of mental illness more than Chinese (Edman & Koon, 2000). Although our results do not describe religious attribution of mental illness, it highlights a cultural difference in how the two ethnic groups perceive mental illness. Mental health professionals and MHL programmes may wish to consider these differences when educating lay people on the causes of mental illness.

Chong *et al*. (2016) found age differences in the same sample with younger adults (18–34 years) having better recognition of mental illnesses than those aged 35 years old and above. Younger adults also show less stigma towards mental illness and higher openness to seeking professional psychological help (Picco *et al*. 2016; Subramaniam *et al*. 2016). Since causal beliefs are related to stigma and help-seeking behaviours (Corrigan, 2004; Reavley & Jorm, 2014*a*), it appears that the age differences found in our sample are congruent with previous studies with older adults (35–49 years) being less likely to endorse psychosocial causes than younger adults (18–34 years). This may reflect the changing views towards mental illness and the possible effect of awareness campaigns in educational settings. Youths may also have more exposure to information regarding mental illness via the internet and social media, leading to the age differences observed.

Other sociodemographic differences include physical causes being less likely to be endorsed by males, homemakers/students/retirees compared with the employed and those with income of $2000 and above compared with those with less than $2000. These differences have not been found in previous research and are hard to interpret. While the results show there is a difference in these groups, awareness campaigns may not necessarily need to target different groups when educating the public about the physical causes of mental illness. The topic should however be breached with all groups on the different causes in order to provide holistic education on mental illness.

The relationship between education and causal beliefs in our sample is similar to those found in Australia. Having a polytechnic diploma (post-secondary diploma) was associated with more endorsement of personality causes compared with those with university level education. Primary and secondary educated participants were also more likely than university educated participants to endorse physical causes. Reavley & Jorm (2014*b*) found that those with Bachelor's level or higher education were less likely to believe in causes other than biogenetic explanations of schizophrenia compared with those with lower education. As causal beliefs are thought to affect help-seeking behaviour (Chen & Mak, 2008; Bhikha *et al*. 2015), public education for MHL should be delivered in a format that is easily understandable for those with less education so that they may understand the causes of mental illness and treatment options.

The participants in our study were more likely to associate particular causal attributions with certain mental illnesses. Physical causes were more likely to be endorsed for depression, OCD and schizophrenia compared with alcohol abuse. Although alcohol abuse is considered a psychiatric condition and is included in the Diagnostic and Statistical Manual of Mental Disorders (4th edition), it is often viewed as being separate from mental illness (Link *et al*. 1999). Those with alcohol related problems are ‘less frequently regarded as mentally ill, are held much more responsible for their condition’ compared with those with substance-unrelated mental illnesses (Schomerus *et al*. 2011). This may account for the difference in attribution of alcohol abuse compared with the other mental illnesses. Of some concern is that participants attributed weakness of personality as a cause of depression and this could imply a negative evaluation of the sufferer as a person and could increase stigma (Jorm *et al*. 1997). Although it is only significantly associated with depression, the notion of a ‘weak personality’ being the cause of mental illnesses should be addressed in interventions to reduce misconceptions about the mentally ill. As for participants being less likely to endorse psychosocial causes for OCD and personality causes for dementia, further research may be needed to explore this phenomenon.

The findings of this study should be considered in view of some limitations. Although the study had a good response rate (71%), the causal beliefs of mental illness and desire for social distance of non-respondents may be significantly different from those who participated in the study. It is difficult to predict how they could have affected the study results. However, the large sample size, good response rate and replicable methodology make the findings easy to compare with those conducted in other countries as well as future studies in the same population.

Overall, our findings elucidated the beliefs about the causes of mental illness in the multi-ethnic population of Singapore and provided comparable data with other studies using the vignette approach to MHL. Being one of the few studies investigating causal beliefs of OCD, alcohol abuse and dementia, it has shed light on some of the beliefs Southeast Asian populations have towards these illnesses. Studies in other cultures and ethnic groups can investigate causal beliefs of these illnesses for comparison. MHL interventions can benefit by addressing the beliefs identified in this study to help demystify the causes of mental illness. It would also be interesting to investigate changes in these beliefs over time and its impact on stigma and help-seeking in the local population. Future research should also focus on developing the causal beliefs scale to include more items for biogenetic causes, supernatural causes as well as other potential beliefs, which have not yet been explored in order to better understand the attributions of mental illness in Asian populations.

## Supplementary material

For supplementary material accompanying this paper visit http://dx.doi.org/10.1017/S2045796017000105. click here to view supplementary material

## Availability of Data and Materials

Data supporting the findings are available upon request. Please contact the corresponding author Mythily Subramaniam (mythily@imh.com.sg) for data availability.
