# Supplementary material for: The Singaporean public beliefs about the causes of mental illness: results from a multi-ethnic population-based study
Source: Epidemiol Psychiatr Sci. 2017 Apr 3;27(4):403–12. doi: 10.1017/S2045796017000105 (PMC6998867; doi:10.1017/S2045796017000105)
Supplement: Supplementary file 1 [file S2045796017000105sup001.docx]

**Supplementary Table 1**

| Items |  | Mean | Std. Deviation | Correlation coefficient |  |  |  |  |  |  |  |  |  |
| --- | --- | --- | --- | --- | --- | --- | --- | --- | --- | --- | --- | --- | --- |
|  |  |  |  | V1 | V2 | V3 | V4 | V5 | V6 | V7 | V8 | V9 | V10 |
| **A virus or other infection** | V1 | .23 | .418 | 1.000 |  |  |  |  |  |  |  |  |  |
| **An allergy or reaction** | V2 | .28 | .450 | .414 | 1.000 |  |  |  |  |  |  |  |  |
| **Everyday problems such as stress, family arguments difficulties at work or financial difficulties** | V3 | .95 | .219 | .009 | .021 | 1.000 |  |  |  |  |  |  |  |
| **The recent death of a close friend or relative** | V4 | .79 | .405 | -.003 | -.041 | .309 | 1.000 |  |  |  |  |  |  |
| **Some recent traumatic event such as a severe traffic accident** | V5 | .81 | .396 | .052 | .001 | .211 | .401 | 1.000 |  |  |  |  |  |
| **Childhood problems such as being badly abused, losing one or both parents when young or coming from a broken home** | V6 | .82 | .388 | .041 | .075 | .269 | .306 | .277 | 1.000 |  |  |  |  |
| **Inherited or genetic or run in the family** | V7 | .63 | .484 | .067 | .058 | .075 | .128 | .154 | .099 | 1.000 |  |  |  |
| **Spirit possession, supernatural causes or black magic** | V8 | .18 | .384 | .138 | .069 | .040 | .099 | .080 | .107 | .163 | 1.000 |  |  |
| **Being a nervous person** | V9 | .73 | .443 | .062 | .106 | .137 | .096 | .144 | .198 | .020 | .054 | 1.000 |  |
| **Having a weak character** | V10 | .70 | .460 | .017 | .098 | .207 | .145 | .152 | .289 | .037 | .037 | .385 | 1.000 |
